# The relationship between adverse childhood experiences and depression: A cross-sectional survey with university students in Botswana

**DOI:** 10.4102/sajpsychiatry.v26i0.1444

**Published:** 2020-11-03

**Authors:** Kennedy Amone-P’Olak, Nkalosang K. Letswai

**Affiliations:** 1 Department of Psychology, Faculty of Humanities, University of the Witwatersrand, Johannesburg, South Africa; 2 Department of Psychology, University of Botswana, Gaborone, Botswana

**Keywords:** adverse childhood experiences, depression, young adults, psychological abuse, Botswana

## Abstract

**Background:**

Adverse childhood experiences (ACEs) are associated with severe life-long negative outcomes, including depression. Particularly in low- and middle-income countries, few studies have been conducted to assess the impact of ACEs.

**Aim:**

To assess the influence of ACEs on depression among young adults.

**Setting:**

Participants were students at a large university in Gaborone, Botswana.

**Methods:**

Using a cross-sectional design, we investigated the associations between ACEs and depression in young adults in Botswana (*n* = 392, mean age = 22.2, ± 2.5, 53.4% female). Bivariate correlation analyses, *t*-tests and analyses of variance (ANOVA) were performed to assess associations and compare ACEs at different levels of depression.

**Results:**

A total of 73% (*n* = 287) reported one or more ACEs, whilst 15% (59) reported five or more ACEs. About 64% (38) of those who reported five or more ACEs were female respondents. Prevalence of specific ACEs ranged from 9.5% (child neglect) to 36.3% (separation and divorce). One in three respondents reported parental separation or divorce, psychological abuse and family dysfunction, whilst 19% (11% moderate and 8% severe) reported significant depressive symptoms. Adverse childhood experiences significantly predicted depression (β = 0.27, 95% confidence interval [CI]: 0.18, 0.37). Respondents at different levels of depression significantly differed on reporting ACEs (*F*_(3, 389)_ = 11.43, *p* < 0.001).

**Conclusion:**

Adverse childhood experiences are highly prevalent and key determinants of depression in young adulthood. A multifaceted and cross-system intervention (e.g. schools, social work, psychological services, health services and law enforcement) is required to protect, prevent and treat survivors of childhood adversity.

## Introduction

Depression amongst young adults in low- and middle-income countries is a growing public health concern. Several factors have been implicated in the development of depressive disorders. Such factors include poverty,^1^ sexual abuse,^2^ conflict,^3^ unemployment^4^ and adverse childhood experiences (ACEs).^5,6,7,8^

Compared with Europe and other Western countries, little attention has been given to ACEs as risk factors of depression in low- and middle-income countries. Yet, ACEs are common and often associated with considerable impairment and form the basis of later mental health problems, such as depression.^7^ Understanding the trajectory and influence of ACEs on health may inform interventions aimed at ending the intergenerational continuity of poor education, unemployment and income inequalities and failure to achieve full health, and social and economic potential of child survivors of ACEs.^9^

Particularly in early adulthood, depression can have devastating consequences, such as school dropout, poor grades, and burden to the criminal justice system, and is a risk factor to alcohol abuse, suicidal behaviours and road traffic accidents.^8,10,11^ Furthermore, depression is the leading cause of years lived with disability (estimated years of life spent in less than full health), especially in the age range of 15–44 years.^8^

In countries such as Botswana, few studies have been carried out on depression amongst young adults, which have shown prevalence rates of severe depression ranging from about 8% to 24% in young adults.^2,12,13,14^

A high density of risk factors exists in Botswana that may put children at risk of adverse childhood maltreatment and abuse that may later lead to depression in young adulthood. For example, Botswana has one of the worst human immunodeficiency virus (HIV) and acquired immunodeficiency syndrome (AIDS) statistics in the world, with a prevalence of 20.3% in the general population aged 15–49 years. In the same age group, the prevalence amongst women is 24.6% and amongst men it stands at 16.2%.^15^

As a result, many children are living with HIV and AIDS or orphaned or looking after significant others with HIV and AIDS. Many of these children are often vulnerable to maltreatment and abuse.

Although Botswana is an upper-middle-income country, poverty persists amongst a considerable section of the population, amongst whom child maltreatment is rife.^16,17^ For example, previous studies have demonstrated that risk factors of childhood adversity included family violence,^18^ drug and substance abuse^19,20,21^ and child abuse.^2,21^ Therefore, these risk factors may be associated with depression in young adults.^18,22^

Although a few studies have examined the prevalence and correlates of depression amongst young adults at universities in Botswana,^2,12,13,14^ no studies have examined the possible influence ACEs on depression.

University students not only experience numerous stressors^23^ but are also vulnerable to depression as a result of ACEs, such as childhood sexual abuse.^2^ Therefore, based on the literature, we hypothesise that young adults who experienced more ACEs are more likely to report more depressive symptoms.

This study assessed the influence of ACEs on depression in a sample of students enrolled in a university in Botswana. Particularly, the study aimed to assess whether different levels of depressive symptomatology will vary depending on the number of ACEs reported. The gender differences in reporting depression and ACEs will be investigated as well.

Embedded within the psychosocial theory of depression, the preponderance of depression in women is partly explained by the fact that women are exposed to a higher density of stressors in childhood, adolescence and adulthood.^24,25^ Another explanation for the higher prevalence of depression amongst women is that women and men respond differently to stressors.^26^ Besides, biological mechanisms (e.g. hormones) have been implicated in modifying the effects of stressors on depression.^27,28^ For these reasons, the gender difference in the relationship between ACEs and depression will be investigated.

## Method

### Design and setting

A cross-sectional survey was conducted with students at one of the premier and largest universities in Botswana. Participants were drawn from a population of young adults aged 18–25 years enrolled for various study programmes at the university. Altogether, 419 participants were requested to participate in this study. However, 10 students declined to participate in the study, whilst 17 provided incomplete information. Finally, data from 392 students were included in the study, representing a 93.6% response rate, 213 (54.3%) of whom were women.

### Measures

Three different categories of measures were used in this study. Firstly, a self-made instrument was used to measure socio-demographic characteristics, such as age, sex, academic performance, place of upbringing and parental educational attainment. Secondly, depressive symptoms were measured using the 21-item Beck’s Depression Inventory II (BDI-II).^29^ Thirdly, the 10-item ACEs questionnaire was used to assess ACEs before 18 years of age.^30,31^

#### Depressive symptoms

The BDI-II^29^ is often used with non-clinical population to screen for symptoms of depression in the past 2 weeks including the day of screening. Examples of depressive symptoms in the BDI-II scale include ‘worthlessness’ with four possible response categories scored as follows: ‘I do not feel I am worthless’ (= 0) through ‘I feel I am utterly worthless’ (= 3).

The scores are summed up (0–63), with higher scores indicative of the severity of depressive symptoms. Scores were categorised as follows: minimal depression (0–13), mild depression (14–19), moderate depression (20–28) and severe depression (29–63).^29^ The BDI-II has been previously used with the same population in Botswana^2,12,13,14^ and South Africa,^32^ with acceptable Cronbach’s alpha reliabilities ranging from α = 0.83 to α = 0.93. For the current study, the internal consistency reliability was acceptable at α = 0.92.

#### Adverse childhood experiences

The 10-item ACEs questionnaire retrospectively measures emotional, physical and sexual abuse, neglect and household dysfunction.^30,31^ Examples of the items on the ACEs questionnaire include ‘were your parents ever separated or divorced?’ and ‘did a parent or other adult in the household often swear at you, insult you, put you down, or humiliate you or act in a way that made you afraid that you might be physically hurt?’

All the items were dichotomously scored ‘yes’ (= 1) and ‘no’ (= 0), with a higher score indicating more adversity. For the present study, the Cronbach’s alpha was acceptable at α = 0.83.

### Procedures

Respondents filled in the questionnaire anonymously in various lecture theatres of the different faculties of the university in the presence of research assistants. The research assistants had obtained prior permission from the lecturers to collect data during their lecture hours. The respondents were students enrolled in various study programmes and were at different levels of their studies. The research assistants explained the purpose of the study, the rights of respondents and obtained written informed consent before distributing the questionnaire. It took about 15 min to complete the questionnaire. Altogether, data from 392 students (54.3% women; *n* = 213) with an average age of 22.2 years (± 2.5, aged 18–25 years) were used in the present study.

### Statistical analyses

Descriptive statistics (e.g., mean, standard deviation [s.d.] and range) were used to compute the socio-demographic characteristics of the respondents, especially age and prevalence rates of ACEs and depressive symptoms.

The results were stratified by gender and tabulated. Pearson’s product–moment correlations analyses and *t*-tests were used to assess associations between variables and gender differences in reporting ACEs and symptoms of depression. Adverse childhood experience was used as the independent variable in a one-way analysis of variance (ANOVA) with categories of depressive symptoms (minimal, mild, moderate and severe) as the dependent variable. The effect sizes of the ACEs on various categories of depressive symptoms were computed using Eta-squared (η^2^). The Tukey’s honestly significant difference Honestly Significant Difference (HSD)-post hoc tests for multiple comparisons were used to compare the mean scores of depressive symptoms at various ACEs thresholds (0-ACEs, 1–2 ACEs, 3–4 ACEs and 5+ACEs).

All statistical analyses were carried out using IBM SPSS statistical software, version 25.0.^33^ Associations with a *p* < 0.05 were considered statistically significant.

### Ethical consideration

Permission to carry out this study was granted by the Institutional Review Board of the University of Botswana. The respondents were guaranteed confidentiality and anonymity, and they were asked not to write anything or put any mark on the questionnaire that would identify them. Finally, after data collection, contact information for psychological support services within the university was made available to the respondents in case they may need help as a result of participating in the study. The telephone numbers for the Psychology Clinic and Counselling Centre, both within the University of Botswana campus, were given to the participants.

## Results

### Socio-demographic characteristics of the study population

The socio-demographic characteristics of the respondents are presented in Table 1. The sample included 392 undergraduate students, 213 (54.3%) of whom were female respondents. Male respondents were significantly older than their female counterparts (Table 1). About 57% (*n* = 224) of the respondents reported that their mothers or maternal guardian had attained vocational or university education, whilst 65% indicated that their fathers or paternal guardian had achieved vocational or university education.

**TABLE 1:** Socio-demographic characteristics and prevalence of adverse childhood experiences and depressive symptoms in the study participants.

Variables	Total				Male				Female				*t*-test
	*M*	*N* or *n*	s.d.	%	*M*	*N* or *n*	s.d.	%	*M*	*N* or *n*	s.d.	%	
**Participants**	-	392		100	-	179	-	45.7	-	213	-	54.3	-
**Age (years)**	22.2	-	2.5	-	22.7		2.5	-	21.6	-	2.4	-	3.98, *p* < 0.01
18–20	-	108	-	27.6	-	31	-	17.3	-	77	-	36.2	-
21–24	-	244	-	62.2	-	122	-	68.2	-	122	-	57.3	-
25–28	-	31	-	7.9	-	23	-	12.8	-	8	-	3.8	-
29–32	-	9	-	2.3	-	3	-	1.7	-	6	-	2.8	-
**Adverse childhood experiences**	2.2		2.1	-	2.1	-	1.9	-	2.3	-	2.2	-	ns
0	-	105	-	26.8	-	48	-	26.8	-	57	-	26.8	-
1	-	83	-	21.2	-	38	-	21.2	-	45	-	21.1	-
2	-	52	-	13.3	-	22	-	12.3	-	30	-	14.1	-
3	-	54	-	13.8	-	28	-	15.6	-	26	-	12.2	-
4	-	39	-	9.9	-	22	-	12.3	-	17	-	8.0	-
≤ 5	-	59	-	15.1	-	21	-	11.8	-	38	-	17.9	-
**Depression**	11.0	-	10.7	-	10.4	-	10.1	-	11.6	-	11.1	-	ns
Minimal	-	266	-	67.9	-	129	-	72.1	-	137	-	64.3	-
Mild	-	52	-	13.3	-	23	-	12.8	-	29	-	13.6	-
Moderate	-	44	-	11.2	-	18	-	10.1	-	26	-	12.2	-
Severe	-	30	-	7.7	-	9	-	5.0	-	21	-	9.9	-

*M*, mean; *N*, total number of participants; *n*, subpopulation; s.d., standard deviation; %, per cent; ns, not significant.

Approximately 73% (*n* = 287) of the respondents reported one or more ACEs, with at least 15% (*n* = 59) of the whole sample reporting five or more adverse experiences. Out of the respondents who reported five or more ACEs, 64% (*n* = 38) were female respondents (Table 1). About one in three respondents reported parental separation, psychological abuse or family dysfunction (Table 2). Furthermore, one in four respondents reported physical abuse.

**TABLE 2:** Reporting adverse childhood experiences stratified by gender (*N* = 392).

S. no.	Adverse childhood experiences	Total Yes		Male Yes		Female Yes		*t*-test (df = 392)
		*n*	%	*n*	%	*n*	%	
1	Were your parents ever separated or divorced?	139	36.3	70	40.9	69	32.5	2.07, *p* < 0.05
2	Did a parent or other adult in the household often swear at you, insult you, put you down or humiliate you? or Act in a way that made you afraid that you might be physically hurt?	137	35.0	57	31.8	80	37.7	ns
3	Did you often feel that … No one in your family loved you or thought you were important or special? or Your family didn’t look out for each other, feel close to each other or support each other?	130	33.4	53	29.9	77	36.3	ns
4	Did a parent or other adult in the household often … push, grab, slap or throw something at you? or Ever hit you so hard that you had marks or were injured?	101	25.8	48	26.8	53	24.9	ns
5	Did you live with anyone who was a problem drinker or alcoholic or who used street drugs?	87	22.3	43	24.2	44	20.7	ns
6	Was a household member depressed or mentally ill or did a household member attempt suicide?	68	17.4	30	16.9	38	17.8	ns
7	Was your mother or stepmother often pushed, grabbed, slapped or had something thrown at her? or Sometimes or often kicked, bitten, hit with a fist or hit with something hard? Ever repeatedly hit over at least a few minutes or threatened with a gun or knife?	57	14.6	22	12.4	35	16.4	ns
8	Did an adult or person at least 5 years older than you ever … touch or fondle you or have you touch their body in a sexual way? or Try to or actually have oral, anal or vaginal sex with you?	55	14.1	19	10.6	36	17.1	2.19, *p* < 0.05
9	Did a household member go to prison?	40	10.2	16	8.9	24	11.3	ns
10	Did you often feel that … you didn’t have enough to eat, had to wear dirty clothes and had no one to protect you? or Your parents were too drunk or high to take care of you or take you to the doctor if you needed it?	37	9.5	16	9.0	21	9.9	ns

*N,* total sample; *n,* subpopulation; %, per cent; df, degrees of freedom; ns, not significant.

Although there was no gender difference in reporting the total number of ACEs, female respondents reported significantly more sexual abuse than their male counterparts, whilst male respondents reported significantly more parental separation and divorce than their female peers (Table 2).

Regarding depressive symptoms, about 19% (*n* = 74) of respondents reported moderate to severe symptoms, with nearly 8% (*n* = 30) in the severe category. Even though there were no overall gender differences in reporting depressive symptoms, more female respondents (*n* = 21, 70%) were in the severe group than their male counterparts (*n* = 9, 30%).

The most commonly reported types of ACEs were divorce or separation (which was reported by about 36% of respondents), psychological abuse (reported by 35%), family dysfunction (reported by 33%) and physical abuse (reported by 26%) (Table 2). Adverse childhood experiences significantly predicted depression (β = 0.27, 95% confidence interval [CI]: 0.18, 0.37).

### Analyses of variance results for total sample

To test the hypothesis that reporting more ACEs varied with different levels of depressive symptoms, a one-way between-subjects ANOVA was conducted with ACEs as the independent variable and symptoms of depression as the outcome variable. Mean scores on depression were compared for the different categories of ACEs: respondents with no history of ACEs, 1–2 ACEs, 3–4 ACEs and ≥ 5 ACEs.

**FIGURE 1:**
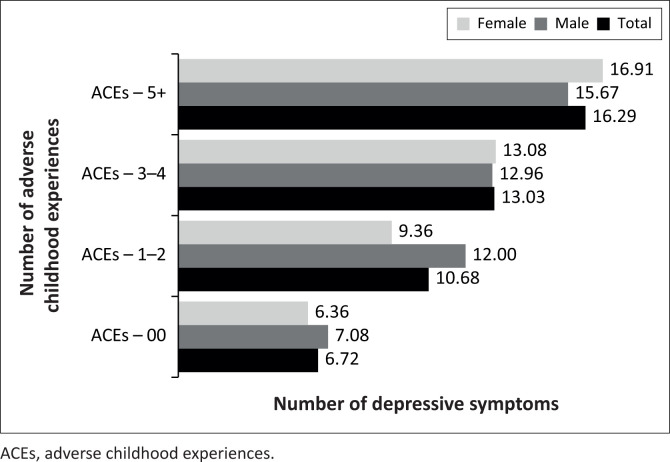
Depressive symptoms stratified by the number of adverse childhood experiences.

The results showed that respondents at different categories of ACEs significantly varied in reporting depressive symptoms (*F*_(3, 388)_ = 12.62, *p* < 0.001, η^2^ = 0.09). The Tukey’s HSD post hoc tests were used to compare the mean scores of depressive symptoms at various ACEs thresholds. Respondents with no history of ACEs (*M* = 6.72, s.d. = 7.42) significantly differed from those with 1–2 ACEs (*M* = 10.68, s.d. = 9.66), 3–4 ACEs (*M* = 13.03, s.d. = 11.22) and ≥ 5 ACEs (*M* = 16.29, s.d. = 13.69). However, respondents who experienced 1–2 ACE events and 3–4 ACE events did not significantly differ in reporting depressive symptoms as were those who experienced 3–4 and ≥ 5 ACEs, suggesting a possible ‘ceiling effect’. The mean scores on depressive symptoms for the different ACE categories are presented in Figure 1. Separate analyses for male and female participants yielded similar results as described here.

### Analyses of variance results for male participants

Mean scores on depression were compared for the different categories of ACEs: respondents with no history of ACEs, 1–2 ACEs, 3–4 ACEs and ≥ 5 ACEs. The results showed that respondents at different categories of ACEs significantly varied in reporting depressive symptoms (*F*_(3, 175)_ = 11.19,*p* < 0.001, η^2^ = 0.11).

The Tukey’s HSD post hoc tests were used to compare the mean scores of depressive symptoms at various ACEs thresholds. Male respondents with no history of ACEs (*M* = 7.08, s.d. = 5.13) significantly differed from those with 1–2 ACEs (*M* = 12.16, s.d. = 9.03), 3–4 ACEs (*M* = 13.00, s.d. = 11.07) and ≥ 5 ACEs (*M* = 15.67, s.d. = 12.90). However, respondents who experienced 1–2 ACE events and 3–4 ACE events did not significantly differ in reporting depressive symptoms as were those who experienced 3–4 and ≥ 5 ACEs, again suggesting a possible ‘ceiling effect’ like in the total score. The mean scores on depressive symptoms for the different ACE categories are presented in Figure 1. The results follow the same pattern as the results of the total sample.

### Analyses of variance results for female participants

Mean scores on depression were compared for the different categories of ACEs: respondents with no history of ACEs, 1–2 ACEs, 3–4 ACEs and ≥ 5 ACEs. The results showed that female respondents at different categories of ACEs significantly varied in reporting depressive symptoms (*F*_(3, 209)_ = 12.55, *p* < 0.001, η^2^ = 0.08).

The Tukey’s HSD post hoc tests were used to compare the mean scores of depressive symptoms at various ACEs thresholds. Respondents with no history of ACEs (*M* = 6.36, s.d. = 5.33) significantly differed from those with 1–2 ACEs (*M* = 9.20, s.d. = 7.76), 3–4 ACEs (*M* = 13.06, s.d. = 10.13) and ≥ 5 ACEs (*M* = 16.91, s.d. = 12.54). However, respondents who experienced 1–2 ACE events and 3–4 ACE events did not significantly differ in reporting depressive symptoms. The mean scores on depressive symptoms for the different ACE categories are presented in Figure 1. Again, the results follow a pattern similar to the results of male participants and the total sample.

## Discussion

Hardly any studies have been carried out in Botswana to assess the associations between a range of interrelated ACEs and depressive symptomatology in young adults. In this study, a considerable number of young adults (73%, *n* = 287) experienced at least one ACE. Out of those who experienced ACEs, one in four experienced four or more adverse events. Separation and divorce, psychological abuse and family dysfunction were the most common ACEs reported in that order by at least one in three respondents (Table 2). Similarly, physical abuse and living with someone who abuses drugs and substances was reported by one in four respondents (Table 2). Overall, there were no gender differences in reporting ACE events except for parental separation and divorce, which was reported by more male than female respondents, and sexual abuse that was reported by more female than male respondents.

Adverse childhood experiences are common and highly prevalent in Botswana, highlighting the importance of child protection in the country. For example, the five most commonly reported ACEs include parental separation and divorce, psychological abuse, family dysfunction, physical abuse and living with someone who abuses drugs or substances (Table 2). Adverse childhood experiences such as family violence, drug and substance abuse and child abuse are widespread in Botswana.^2,18,19,20,21^ Similarly, national statistics indicate that only 25.3% of couples are married, 27.3% are cohabiting and the rest are never married, separated and/or divorced or are widowed.^34^ Furthermore, seven out of every 10 children in Botswana come from families with only one parent, often the mother.^35^ Apart from the bleak family statistics in Botswana, the HIV and AIDS epidemic has ravaged the country for a long time, leaving numerous children orphaned. These family environments are fertile grounds for childhood adversity, often associated with long-term mental health problems^5,6,7,8^ and diminished life opportunities in education, employment and income potentials that may span many generations to come.^9,36,37,38^

Reporting more ACE events was associated with an increased severity of depression. Markedly, respondents who experienced 1–2 and 3–4 ACE events did not differ in their levels of depressive symptomatology compared to those who experienced 3–4 and five or more ACE events. This finding may indicate a ‘ceiling effect’ whereby experiencing more ACE events will surpass any vulnerability over and above a nominal number of depressive symptomatology. The possible ‘ceiling effect’ is consistent with the cumulative stress hypothesis in which the accumulation of adverse life events is associated with health problems, such as depression.^39,40^

Evidence from neurobiological and epidemiological studies links childhood adversity to changes in brain structures, function and stress response with long-term adverse consequences.^41^ For instance, stress, such as those resulting from ACEs, inhibits the growth of new neurons, brain functions and contributes to dysregulation of the hypothalamic–pituitary–adrenocortical (HPA) system.^42,43,44,45,46^ All these may have life-long consequences for education, employment, income and other life opportunities.^9^

Furthermore, a high density of ACEs is located in specific subpopulations, such as those coming from dysfunctional family background and living in poverty (e.g., rural dwellers, single mothers, and people afflicted by diseases such as HIV and AIDS and tuberculosis). The findings that separation, divorce, psychological and physical abuse, family dysfunction and drug and substance abuse were more prevalent than other ACEs confirm the findings of previous studies. For example, in Botswana, 70% of children come from a family background with a single parent, usually mothers.^35^ It is widely recognised that more than half of the single mothers and female-headed households live in abject poverty compared with a quarter of their male counterparts.^35^ In addition, it may be important to consider access to protective factors such as social support and resilience factors that may cushion against the adverse effects for survivors of ACEs. For example, individual (e.g., temperament and disposition), family (e.g., attachment and care) and community (e.g., peer relations and community support) help to build resilience.^47^ It is therefore possible that survivors of ACEs with such support are likely to thrive in the aftermath of ACEs more than those without such support. Lastly, the results of this study are a valuable contribution to the literature and confirm previous findings within Botswana.^2,21^

### Limitations and strengths

The results of this study should be carefully interpreted because of several limitations. Firstly, ACEs in this study were reported retrospectively and as such the memory of these events may be inaccurate as suggested in previous studies.^48^ Secondly, ACEs such as sexual abuse or physical abuse are sensitive and emotive subjects that may be hard to report. Consequently, this might have led to under-reporting of the ACEs. Thirdly, the sample in this study comprised not only a heterogeneous group within the university but also an exclusive group that can attend university and may not be representative of the wider community, thus limiting the generalisability of the findings. Fourthly, ACEs are a static one-time category that does not include all types of early childhood adversity that children in low- and middle-income countries experience. For example, many children may be orphaned by diseases such as HIV and AIDS or have experienced bullying at school or domestic and/or community violence. Therefore, the list of adversities included in the ACE scale is by no means exhaustive. Fifthly, the design of this study was a cross-sectional survey. Consequently, it is difficult to infer causality, although the ACEs undoubtedly came before the assessment of depressive symptoms. Similarly, this study did not consider the possibility of previous access to social and psychological support and other community resources (e.g., extended family and social workers) likely to buffer the toxic effects of ACEs and subsequently improve the life outcomes and opportunities of survivors of ACEs. Finally, drug and substance use in young adults has also been associated with mental health problems such as depression.

###  Implications

Despite the limitations outlined here, the findings of this study have several implications for research, policy and practice. Regarding research, there is an urgent need for longitudinal studies with a larger sample size covering children from diverse backgrounds. A longitudinal study will assess the general conditions of children and unravel the long-term consequences of ACEs in low- and middle-income countries, such as Botswana. Moreover, a prospective longitudinal study will help to identify children and families at risk of ACEs to inform interventions in order to mitigate the toxic effects of ACEs. Furthermore, child protection policies should be strengthened and enforced. Social workers, psychologists and health workers should be employed in all villages and communities to continuously evaluate the welfare of children and provide remedies. For example, policies such as creating a children’s bureau to assess all children every 6 months or so for their health and well-being until they are 16 years of age should be adopted. Such a bureau will help identify and tackle any ACEs early enough and create an environment that ensures child protection. Furthermore, future studies should consider the influence of poverty when studying the relationship between early adversity and depression.

## Conclusion

The findings of the current study demonstrate that experiencing adversity during childhood is significantly associated with reporting depressive symptomatology. Several studies have shown that the effects of childhood adversity are associated with later mental and physical health problems and diminished life opportunities that span over generations. Understanding factors that lock families in the path of early childhood adversity is vital for unleashing the full potential and future opportunities of children. A multifaceted cross-system intervention (e.g., schools, social work, psychological services, health services and law enforcement) to protect, prevent and treat survivors is required to meet the developmental and mental health needs of young adults exposed to ACEs.
